# Duration of resuscitation, regain of consciousness and histopathological severity of hypoxic-ischemic encephalopathy after cardiac arrest

**DOI:** 10.1016/j.resplu.2025.100945

**Published:** 2025-03-24

**Authors:** Christian Endisch, Katharina Millard, Sandra Preuß, Werner Stenzel, Jens Nee, Christian Storm, Christoph J. Ploner, Christoph Leithner

**Affiliations:** a Department of Neurology, AG Emergency and Critical Care Neurology, Campus Virchow Klinikum, Charité Universitätsmedizin Berlin, Augustenburger Platz 1, 13353 Berlin, Germany; b Department of Cardiology and Angiology, Charité Campus Mitte, Charité Universitätsmedizin Berlin, Charitéplatz 1, 10117 Berlin, Germany; c Department of Neuropathology, Charité Campus Mitte, Charité Universitätsmedizin Berlin, Charitéplatz 1, 10117 Berlin, Germany; d Telehealth Competence Center GmbH, Humboldtstraße 67a, 22083 Hamburg, Germany

**Keywords:** Cardiac arrest, Regain of consciousness, tROSC, Hypoxic-ischemic encephalopathy, Brain autopsy, Postmortem, Prognosis

## Abstract

**Purpose:**

To study the histopathologically quantified severity of hypoxic-ischemic encephalopathy (HIE) in deceased cardiac arrest unbiased by death causes and correlated with demographic parameters.

**Methods:**

We conducted a retrospective, single-centre study including cardiac arrest patients with postmortem brain autopsies. Using the selective eosinophilic neuronal death (SEND), the histopathological severity of HIE was quantified in the cerebral neocortex, hippocampus, basal ganglia, cerebellum, and brainstem, and correlated with demographic parameters.

**Results:**

We included 319 patients with a median time of return from cardiac arrest to spontaneous circulation (tROSC) of 10 min, of whom 62(19.4%) had a regain of consciousness (RoC) before death. The tROSC was significantly correlated with the SEND in all brain regions (*p* < 0.05, Spearman’s rho = 0.14 to 0.29). The SEND in the neocortex, hippocampus, and basal ganglia was significantly correlated with RoC (*p* < 0.05, Spearman’s rho = −0.25 to −0.11). In 9 patients with tROSCs less than 1 min, all had a brainstem SEND less than 30%, and 8(88.9%) had neocortical SEND less than 30%. Among 69 patients with tROSCs greater than 20 min, 47.8–82.6% showed a SEND less than 30% across brain regions.

**Conclusions:**

We found less SEND and RoC was more likely in patients with shorter tROSCs. A tROSC less than 1 min was mostly associated with SEND less than 30% in all brain regions. Prolonged resuscitations with tROSCs greater than 20 min did not exclude a SEND less than 30% in a relevant proportion of patients. Future histopathological studies are warranted to investigate the impact of modifiable clinical parameters on the severity of HIE.

## Introduction

Cardiac arrest (CA) causes global ischemia with a cessation of cerebral blood flow, leading to a sudden loss of consciousness.^1^, ^2^, ^3^ Without the rapid initiation of effective resuscitation, during which cerebral blood flow is maintained at low-flow level and hypoxia may occur, patients may develop irreversible injury in some brain regions within 1–2 min.^1^, ^2^, ^4^ In cases where resuscitation successfully restores spontaneous circulation, patients frequently remain comatose and are treated on an intensive care unit (ICU) to prevent secondary brain injury.^5^, ^6^, ^7^

In these comatose patients, the severity of hypoxic-ischemic encephalopathy (HIE) is the main determinant for the overall clinical outcome and is related to patient demographics and modifiable clinical parameters during resuscitation.^8^, ^9^ The association between these demographics and modifiable clinical parameters has been previously investigated in large-sized clinical studies and has led to a substantial improvement in resuscitation care.^10^ However, a limitation of these studies is the use of clinical outcomes, such as mortality or poor outcome, for instance, assessed by the cerebral performance category (CPC).^5^, ^11^ Clinical outcome scores cannot differentiate between death from severe HIE and extracerebral complications, occurring frequently in comatose CA patients without HIE.^12^, ^13^, ^14^ Furthermore, withdrawal of life-sustaining therapy (WLST) in comatose patients with an erroneous prediction of severe HIE can potentially cause a self-fulfilling prophecy.^15^ Overall, this results in a clinical heterogeneity of deceased CA patients challenging the neuroprognostication of HIE and validation of therapeutic interventions. 5, 6, 7, 8, 9

A more granular approach to study the impact of demographic parameters on HIE is the histopathological quantification of the severity of HIE in CA patients who underwent a postmortem brain autopsy after death.^4^, ^16^, ^17^, ^18^, ^19^, ^20^, ^21^ This autopsy-based approach bypasses clinical confounders and focuses on the histopathological severity of HIE, largely unbiased by the clinical factors leading to death.^12^, ^13^, ^14^ Surprisingly, despite unique histopathological insights into the pathophysiology of HIE, large-sized studies on the relationship between demographics of CA and the histopathological severity of HIE are hitherto lacking.

Therefore, our study aimed to investigate the association between the histopathological severity of HIE and demographics, with a focus on CA patients who either had a short or prolonged time of return of spontaneous circulation (tROSC), and on patients with a temporary regain of consciousness (RoC) before death.

## Material and methods

### Study setting and population

The ethical committee of the Charité University Hospital, Berlin, Germany, approved the study (EA2/007/16) and waived the need for patient consent. In a retrospective study, we enrolled adult patients who were resuscitated from CA, admitted to the ICU, and in whom a postmortem brain autopsy was conducted after death. The enrolment period was from January 2008 to May 2017. Demographics were collected following the Utstein style, which included whether patients temporarily regained consciousness before death.^22^, ^23^ Our institution adhered to the international guidelines of post-resuscitation care including temperature management with active cooling devices for 24 h and a multimodal neuroprognostication in decisions on withdrawal of life sustaining therapies (WLST).^24^, ^25^

### Histopathological quantification of the severity of HIE

We microscopically re-evaluated and quantified the severity of HIE in postmortem brain autopsies applying the selective eosinophilic neuronal death (SEND) classification, which has been previously described in detail.^16^, ^17^, ^20^, ^21^ The SEND classification was established by Björklund et al.^17^ and has been subsequently corroborated.^16^, ^18^, ^20^, ^21^, ^26^ Briefly, the SEND classification uses the histopathological phenomenon that selective neuronal death can be microscopically seen in haematoxylin-eosin staining as eosinophilic neurons. The underlying pathomechanism of SEND is cerebral reperfusion after successful resuscitation from CA, in contrast to neuronal necrosis that occurs in patients without return of cerebral reperfusion.^1^, ^4^, ^5^, ^17^

We microscopically quantified the SEND from different brain regions and allocated patients to five groups: 0% SEND 0, less than 30% (SEND 1), 30–60% (SEND 2), 60–90% (SEND 3), or greater than 90% (SEND 4). The analysed brain regions included the cerebral neocortex, hippocampus, basal ganglia, cerebellum, and brainstem. Patients without histopathology from all five brain regions were excluded from further analyses. All histopathological analyses were conducted blinded from clinical data. A detailed analysis of the histopathological patterns of HIE will be discussed in a separate paper.

### Statistical analyses

We presented the baseline demographics as mean and/or median with interquartile ranges (IQR), and absolute numbers with percentages as appropriate. We analysed the correlation between the SEND, tROSC, and RoC using the Spearman’s rank correlation. A p-value less than 0.05 was considered statistically significant. We used descriptive statistics to analyse the association between the SEND and baseline demographics, and visualized the results with heatmaps. We employed custom-written MATLAB scripts (The MathWorks, R2019b) for all data analyses.

## Results

### Demographics

We enrolled 350 deceased CA patients who underwent a postmortem brain autopsy, of whom 31 (8.9%) patients were excluded because of incomplete histopathology. The baseline demographics of the 319 included patients are shown in Table 1, stratified by the RoC and the duration of tROSC in 232 patients with available times of tROSC.

**Table 1:** **Baseline demographics. The patient demographics of the 319 included patients, stratified by regain of consciousness and duration of resuscitation.** IQR – inter-quartile range, OHCA – out-of-hospital cardiac arrest, CA – cardiac arrest, tROSC – time from cardiac arrest to spontaneous circulation, ICU – intensive care unit, WLST – withdrawal of life-sustaining treatment, SEND – selective eosinophilic neuronal death. *Missing data for shockable initial rhythm (n = 48), cardiac cause of CA (n = 61), and tROSC (n = 87). The results of the first column will be discussed in a separate study focusing in detail on the histopathological patterns of HIE.

Patient demographics	All patients (n = 319)	Regain of consciousness (n = 62)	No regain of consciousness (n = 257)	tROSC less than 1 min (n = 9)	tROSC 1–5 min (n = 68)	tROSC 6–20 min (n = 86)	tROSC greater than 20 min (n = 69)
**Gender, female (n, %)**	123 (38.6)	24 (38.7)	99 (38.5)	2 (22.2)	32 (47.1)	27 (31.4)	27 (39.1)
**Age, years (median, IQR)**	68 (58–76)	68 (58–77)	68 (58–75)	78 (60–80)	70 (59–79)	67 (58–77)	65 (58–73)
**OHCA, (n, %)**	262 (82.1)	57 (91.9)	205 (79.8)	9 (100)	66 (97.1)	70 (81.4)	41 (59.4)
**Shockable initial rhythm, (n, %)**	68 (25.1)	15 (26.3)	53 (24.8)	0 (0)	12 (23.5)	24 (31.6)	20 (35.1)
**Cardiac cause of CA, (n, %)**	83 (32.2)	32 (38.5)	206 (30.6)	4 (66.7)	12 (23.5)	24 (31.6)	20 (35.1)
**tROSC,** **minutes, (median, IQR)**	10 (5–25)	5 (1–10)	15 (5–30)	0.5 (0.5–0.5)	4 (2–5)	14 (10–15)	30 (27–45)
**Temperature control, (n, %)**	85 (26.7)	11 (17.7)	74 (28.8)	0 (0)	6 (8.8)	25 (29.1)	35 (50.7)
***Regain of consciousness before death, (n, %)***	62 (19.4)	62 (100)	0 (0)	8 (88.9)	21 (30.9)	18 (20.9)	3 (4.4)
***Second CA with a resuscitation during the ICU stay, (n, %)***	140 (43.9)	31 (50)	109 (42.4)	5 (55.6)	17 (25)	28 (32.6)	37 (53.6)
**Length of ICU stay,** **day (median, IQR)**	2 (1–8)	23 (9–51)	1 (0–3)	9 (6–25)	3 (1–21)	3 (0–8)	2 (0.8–4)
***WLST, (n, %)***	139 (43.6)	34 (54.8)	105 (40.9)	5 (55.6)	37 (54.4)	38 (44.2)	27 (39.1)
***SEND in the cerebral neocortex, (mean, IQR)***	1.4 (1.0–2.0)	1.0 (0–1.0)	1.5 (1.0–2.0)	0.9 (0.8–1.0)	1.3 (1.0–1.0)	1.4 (1.0–2.0)	1.6 (1.0–2.3)
***SEND in the hippocampus, (mean, IQR)***	1.8 (1.0–3.0)	1.5 (1.0–2.0)	1.9 (1.0–3.0)	1.2 (1.0–1.3)	1.5 (1.0–1.5)	1.8 (1.0–3.0)	2.2 (1.0–4.0)
***SEND in the basal ganglia, (mean, IQR)***	1.1 (0–1.0)	0.7 (0–1.0)	1.2 (1.0–1.0)	0.8 (0–1.0)	0.8 (0–1.0)	1.1 (0–1.0)	1.5 (1.0–2.0)
***SEND in the cerebellar cortex, (mean, IQR)***	1.4 (0–2.0)	1.1 (0–1.0)	1.5 (0–2.0)	0.3 (0–1.0)	1.1 (0–1.0)	1.6 (1.0–3.0)	1.7 (0–3.0)
***SEND in the brainstem, (mean, IQR)***	0.9 (0–1.0)	0.7 (0–1.0)	1.0 (0–1.0)	0.6 (0–1.0)	0.7 (0–1.0)	0.9 (0–1.0)	1.0 (0–1.0)

### SEND and tROSC

**Fig. 1:**
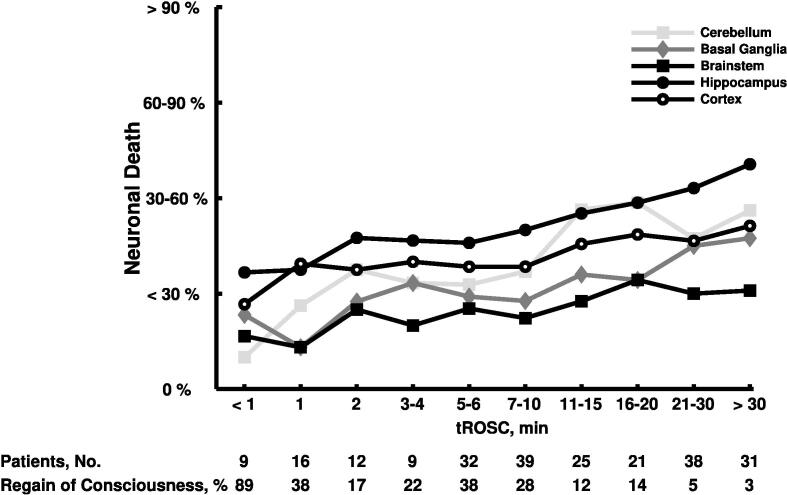
**The association between the selective eosinophilic neuronal death and the time of return from cardiac arrest to spontaneous circulation.** Of 232 patients with available times of return from cardiac arrest to spontaneous circulation, the selective eosinophilic neuronal death is shown as the mean for each investigated brain regions and timepoint. tROSC – time of return from cardiac arrest to spontaneous circulation.

In all five brain regions, longer tROSCs were significantly correlated with an increase in SEND, as shown in Fig. 1 (neocortex: Spearman’s rho = 0.14, *p* = 0.04; hippocampus: Spearman’s rho = 0.23, *p* < 0.001; basal ganglia: Spearman’s rho = 0.29, *p* < 0.001; cerebellum: Spearman’s rho = 0.24, *p* < 0.001; brainstem: Spearman’s rho = 0.14, *p* = 0.03). The hippocampal SEND was higher compared to the neocortical SEND for all tROSCs longer than 1 min. Of all brain regions, the brainstem had the lowest SEND, which increased with longer tROSCs. There was a significant negative correlation between length of tROSC and the proportion of patients with RoC before death (Spearman’s rho = −0.36, *p* < 0.001).

The mean SEND in the neocortex was 0.9 (IQR 0.8–1.0), 1.3 (IQR 1–1), and 1.6 (IQR 1–2), respectively, in patients with a tROSC less than 1 min, 5–6 min, and 21–30 min, respectively. For the same tROSCs, the mean SEND in the hippocampus was 1.2 (IQR 1–1.3), 1.5 (IQR 1–2), and 2.1 (IQR 1–4), respectively, and 0.6 (IQR 0–1), 0.8 (IQR 0–1), and 1.0 (IQR 0–1), respectively, in the brainstem.

In 9 patients with a tROSC less than 1 min, all had a SEND of 0 or 1 in the brainstem, and 8 (88.9%) patients a neocortical SEND of 0 or 1 (Fig. 3A). None of these 9 patients had a SEND of 4 in any brain region.

Surprisingly, of 69 patients with a tROSC longer than 20 min, 45 (65.2%) patients had a SEND of 0 or 1 in the neocortex, 33 (47.8%) in hippocampus, 45 (65.2%) in basal ganglia, 40 (58.0%) in cerebellum, and 57 (82.6%) patients in brainstem, respectively. Conversely, a SEND of 4 in the neocortex, hippocampus, basal ganglia, cerebellum, and the brainstem was found in 10 (14.5%), 21 (30.4%), 8 (11.6%), 16 (23.2%), and 5 (7.3%) patients, respectively. 12 (17.4%) of 69 patients with a tROSC longer than 20 min, had a SEND of 0 or 1 in the brainstem, but a neocortical SEND greater than 1.

### SEND and RoC

We found that 62 (19.4%) of 319 patients regained consciousness before death, of whom 53 (85.5%) had a neocortical SEND of 0 or 1. Death causes of 61 patients with RoC was cardiac in 15 (24.2%), sepsis/multi-organ-failure in 32 (51.6%), brain injury in 4 (6.5%), and other causes in 11 (17.7%) patients.

**Fig. 2:**
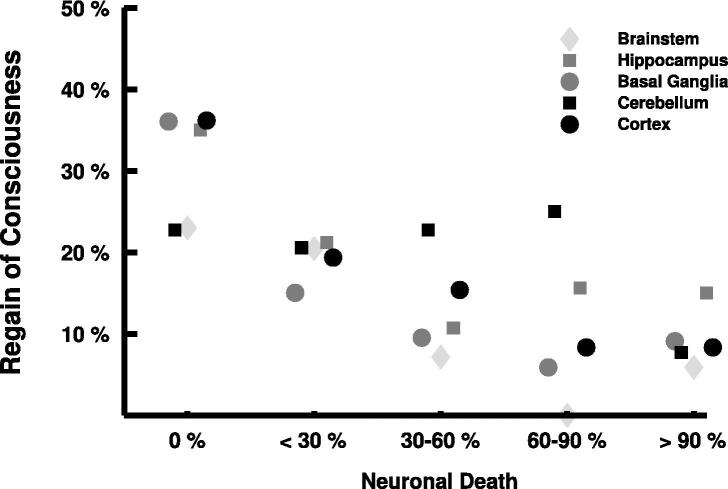
**The association between the selective eosinophilic neuronal death and the regain of consciousness.** This figure illustrates the association between the selective eosinophilic neuronal death (SEND) and the mean percentage of patients with temporary regain of consciousness before death.

**Fig. 3:**
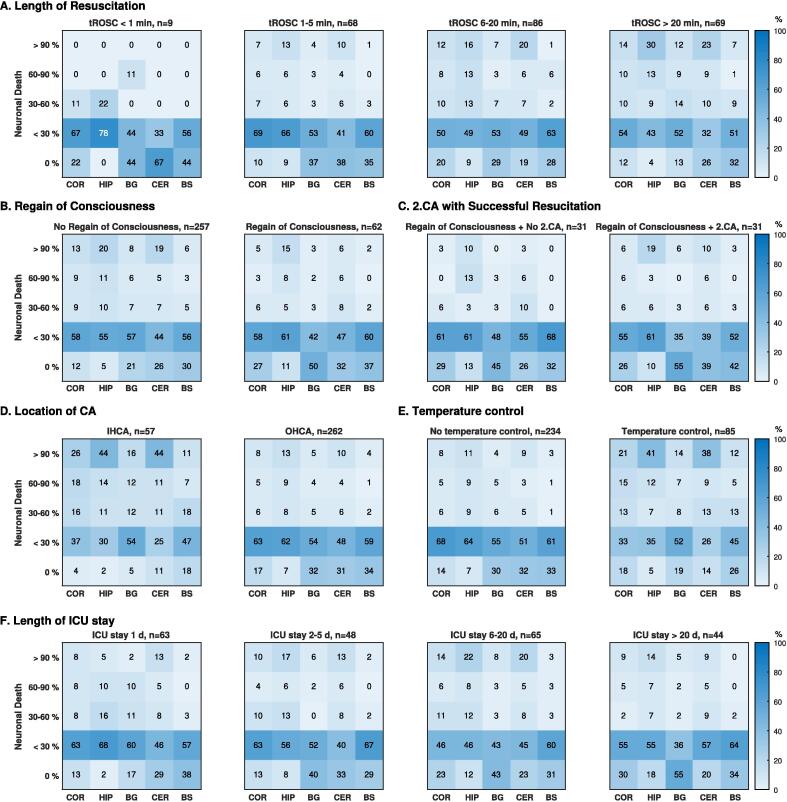
**The distribution of the selective eosinophilic neuronal death stratified according to the baseline parameter.** This figure shows the distribution of the selective eosinophilic neuronal death (SEND) stratified according the relevant baseline parameters (A – E). Using heatmaps, coloured fields with rounded percentages are shown. A: Length of the resuscitation. B: Regain of consciousness before death. C: Regain of consciousness and influence of a second cardiac arrest with successful resuscitation. D: Location of the cardia arrest. E: Application of temperature control. F: Length of the stay on the intensive care unit. CA – Cardiac arrest, IHCA – in-of-hospital cardiac arrest, OHCA – out-of-hospital cardiac arrest, tROSC – time from cardiac arrest to spontaneous circulation.

Fig. 2 shows the significant negative correlation between a decrease in the proportion of patients with RoC before death and an increase in SEND in the cortex (Spearman’s rho = −0.19, *p* < 0.001), hippocampus (Spearman’s rho = −0.11, *p* = 0.04), and basal ganglia (Spearman’s rho = −0.25, *p* < 0.001). In the cerebellum (Spearman’s rho = −0.09, *p* = 0.10) and brainstem (Spearman’s rho = −0.11, *p* = 0.06) this correlation was statistically not significant. Fig. 3B shows the distribution of HIE for the different brain regions stratified by RoC.

Of 47 patients with a neocortical SEND of 0, 17 (36.2%) patients had a RoC, compared to 3 (8.3%) of 36 patients with a neocortical SEND of 4. The median ICU duration of 17 patients with a neocortical SEND of 0 and RoC was 27 days (IQR 21–91), in contrast to 1 day (IQR 0–4) in 30 patients, who never regained consciousness before death. In 18 (60.0%) of 30 patients with neocortical SEND 0, who never regained consciousness, there was a cardiac cause of death.

RoC before death was found in 7 (35.1%) of 20 patients with a SEND of 0 in the hippocampus, 31 (36.1%) of 86 patients with a SEND of 0 in basal ganglia, and 20 (22.7%) of 88 patients with a SEND of 0 in cerebellum, respectively. Of 100 patients with a SEND of 0 in the brainstem, 23 (23.0%) patients had RoC, in contrast to 1 (5.8%) of 17 patients with a SEND of 4 in the brainstem. In 31 patients with RoC, who did not have a second CA with resuscitation during the ICU stay, none had a SEND greater than 1 in the brainstem or greater than 2 in the neocortex, but 7 (22.6%) had a hippocampal SEND greater than 2 (Fig. 3C).

### SEND and baseline demographics

We found that 57 patients with an in-hospital-CA had higher SEND in all regions, compared to 262 patients with an out-of-hospital-CA (Fig. 3D). The SEND was greater in all regions of 85 (26.6%) patients, who were treated with temperature control, compared to 234 (73.4%) patients without temperature control. The median tROSC in patients with temperature control was 24 min (IQR 15–40), compared with a median tROSC of 10 min (IQR 4–20) in patients without temperature control (Fig. 3E). Fig. 3F shows that there was no relevant association between the duration of the ICU stay and the distribution of SEND. In 44 patients with an ICU stay longer than 20 days, 37 (84.1%) had a neocortical SEND of 0 or 1. In contrast to 257 patients who never regained consciousness before death (Fig. 3B), 62 patients with RoC showed less SEND in all brain regions. Specifically, 60 (96.8%) patients had a SEND of 0 or 1 in the brainstem, while a hippocampal SEND greater than 1 was found in 17 (27.4%) patients, compared to a cortical SEND greater than 1 in 9 (14.5%) patients. Among 31 patients with RoC, who did not have a second CA with resuscitation during the ICU stay, only 1 (3.2%) had a neocortical SEND greater than 1, compared with 7 (22.6%) patients with a hippocampal SEND greater than 1. (Fig. 3C).

## Discussion

Our study has several main findings. Firstly, we found a positive correlation between tROSC and histopathological severity of HIE in all brain regions. Secondly, patients with very short tROSC of less than one minute had no or only mild histopathological HIE. Thirdly, despite tROSC of longer than 20 min, many patients had no or only mild HIE in all brain regions, indicating that severe HIE is not a necessary consequence of prolonged resuscitation. Fourthly, the proportion of patients with a temporary RoC decreased with a higher SEND. Fifthly, one fourth of patients with temporary RoC had severe HIE in hippocampus, but severe HIE was rare in other brain regions in these patients.

The positive correlation of the duration of resuscitation with histopathological severity of HIE is an expected finding and in line with clinical studies, in which longer tROSCs are correlated with a higher likelihood of mortality and poor outcome.^6^, ^24^, ^27^, ^28^, ^29^ Corroborating previous postmortem studies,^16^, ^17^, ^20^, ^21^ we found that a longer tROSC is associated with a higher SEND caused by hypoxic-ischemic pathomechanisms in all brain regions, with hippocampus being the most susceptible region with the highest SEND.^1^, ^4^, ^5^, ^17^

Among patients with a tROSC less than one minute, all patients had a SEND of 0 or 1 in the brainstem and most patients a SEND of 0 or 1 in all other brain regions. Although the absolute number of patients with tROSC less than one minute was low in our study, this indicates likely absence of severe histopathological HIE in comatose patients with such short durations of resuscitation in line with clinical studies.^28^, ^29^, ^30^

In patients with a tROSC greater than 20 min, 82.6% had no or mild HIE in the brainstem and 47.8–65.2% no or mild HIE in the other brain regions. Although these proportions are likely altered by the selection bias of autopsy in our cohort, they underscore that even in prolonged resuscitation, the brain can survive without severe HIE.^28^, ^29^ Importantly, several modifiable factors such as bystander CPR rate and efficacy of resuscitation contribute to the final severity of HIE.^5^, ^31^, ^32^ Therefore, in patients with prolonged resuscitations, the decision to stop resuscitation should not solely rely on the duration of resuscitation,^31^, ^33^ especially since the improved availability of extracorporeal life supports.^34^

Previous clinical studies found a lower proportion of survival and good neurological outcome,^35^, ^36^, ^37^ compared to the high proportion of no or mild histopathological HIE of our deceased patients. One likely explanation is a selection bias: Only a small proportion of deceased CA patients were investigated with brain autopsy and it could be that those with no or mild HIE are more likely to undergo a brain autopsy compared to those with a severe HIE. Another explanation is extracerebral causes leading to death in comatose CA patients with no or mild HIE.^12^, ^13^, ^14^, ^16^, ^21^

In a study on the best clinical outcome before death in CA patients, only 4.2% regained consciousness before death.^13^ In comparison, we found that 19.4% of patients regained consciousness before death. Again, this difference is likely explained by a selection bias for conducting postmortem brain autopsies, potentially more frequently in CA patients with anticipated good outcome, but unexpected death. The prevalence of RoC has unfortunately not been reported in previous histopathological studies,^17^, ^18^, ^19^, ^20^ but it varied from 12.8 to 23.3% in our previous own histopathological studies.^16^, ^21^

On the first glance, it seems surprising that among patients with a neocortical SEND of 0, only 17 (36.2%) patients regained consciousness. However, patients without HIE frequently die from extracerebral causes of death, which have the highest incidence in the early post-resuscitation phase.^12^, ^14^, ^38^ In line with this, the median length of ICU stay was lower in neocortical SEND 0 patients, who did not regain consciousness, potentially because early death precluded RoC. Most of these patients died from cardiac death causes, compared to patients with a RoC.

Moreover, a second CA with resuscitation during the ICU may lead to histopathologically severe HIE in patients with RoC before the second CA.^39^ Therefore, in an additional analysis, we excluded a second CA with resuscitation. In this cohort we identified no patients with RoC who had neocortical SEND greater than 2. These histopathological findings corroborate previous neuroimaging studies,^40^, ^41^, ^42^ confirming that the neocortex needs to be structurally intact to a sufficient degree for patients to regain consciousness.^42^

In contrast, in our cohort around one fourth of patients with RoC had isolated severe HIE in the hippocampus. This underscores that the hippocampus is the most vulnerable brain region for neuronal death during CA and that RoC is possible in patients with severe hippocampal HIE.

In the brainstem, the ascending reticular activating system originates, which is essential for the functional re-emergence of arousal in comatose patients.^42^, ^43^ Not surprisingly, we found no patient, who regained consciousness with a SEND greater than 1 in the brainstem, following the exclusion of a second CA with resuscitation as a confounder. The brainstem is the most resilient brain region to oxygen-deprivation.^4^, ^17^, ^44^ In line with previous postmortem studies,^16^, ^17^, ^18^, ^19^, ^20^, ^21^, ^45^ the brainstem was the region with the lowest SEND across different durations of tROSC in our study.

Our results show that postmortem brain autopsies provide a valuable, unfortunately currently underutilised, additional endpoint for future clinical trials and observational studies. Of course, autopsies cannot be obtained in all deceased CA patients, for instance due to the high procedural costs and the refusal of next of kin. However, in a relevant subgroup of patients, postmortem brain autopsies enable the quantification of severity of HIE, unbiased from clinical confounders and WLST decisions, which could have caused a poor clinical outcome despite absence of severe HIE. To utilise postmortem brain autopsies from clinical routine, we suggest to integrate the histopathological HIE severity as an additional outcome measure in future CA studies.

## Limitations

The limitations of our study include the monocentric and retrospective design. As only few deceased CA patients underwent brain autopsies, a selection bias needs to be considered. Due to the protracted enrolment period, postresuscitation treatment was adopted following the recommendations of international guidelines. Future studies should therefore aim to increase the proportion of brain autopsies and shorten the enrolment period. In some investigated subgroups, the absolute patient numbers were small, limiting conclusions. Documentation of some demographic and CA characteristics, especially the tROSC may be imprecise in clinical routine. The absence of histopathological HIE evaluated by SEND does not necessarily prove clinical absence of HIE if the patient had survived in the long-term. Following WLST a prolonged dying process with cerebral hypoperfusion may contribute to secondary HIE. The histopathological characteristics of this process have not been evaluated in detail yet and should therefore be analysed in future studies. Finally, our histopathological analyses required microscopical expertise in rating the SEND classification. Although a previous study has shown excellent inter-rater reliability of the SEND classification,^26^ we did not specifically study this potential confounder. Additionally, the rating of SEND classification relied on human microscopy without using of automated cell counting and classifiers. In future histopathological studies, an automated and machine-learning-based classification of HIE may be used to enable an exact numeric cell detection of viable and hypoxic-ischemic neurons.

## Conclusions

In a large postmortem brain autopsy study, we found a positive correlation of duration of resuscitation with histopathological severity of HIE. Most patients with a tROSC less than 1 min had no or mild histopathological HIE in all brain regions. A relevant proportion of patients with a tROSC longer than 20 min showed no or mild HIE. One fourth of patients with temporary RoC had severe histopathological HIE in hippocampus, but severe HIE war rare in other brain regions in these patients.

## CRediT authorship contribution statement

**Christian Endisch:** Writing – review & editing, Writing – original draft, Visualization, Validation, Supervision, Software, Resources, Project administration, Methodology, Investigation, Funding acquisition, Formal analysis, Data curation, Conceptualization. **Katharina Millard:** Writing – review & editing, Writing – original draft, Visualization, Validation, Methodology, Investigation, Formal analysis, Data curation. **Sandra Preuß:** Writing – review & editing, Writing – original draft, Validation, Project administration, Methodology, Investigation, Formal analysis, Data curation. **Werner Stenzel:** Writing – review & editing, Writing – original draft, Project administration, Data curation. **Jens Nee:** Writing – review & editing, Writing – original draft, Project administration, Data curation. **Christian Storm:** Writing – review & editing, Writing – original draft, Visualization, Validation, Supervision, Software, Resources, Project administration, Methodology, Investigation, Funding acquisition, Formal analysis, Data curation, Conceptualization. **Christoph J. Ploner:** Writing – review & editing, Writing – original draft. **Christoph Leithner:** Writing – review & editing, Writing – original draft, Methodology, Investigation, Funding acquisition, Data curation, Conceptualization.

## Declaration of competing interest

The ZOLL Foundation supported CE with a research fellowship to conduct this study. The Laerdal Foundation and the Berlin Institute of Heath Clinical Fellow Program supported CL. No conflicts of interests are reported by the other authors. The research grants had no role in the study concept, data collection and analysis, decision to publish, or manuscript preparation. The authors have the sole responsibility for the study design and study conduction, all analyses, drafting and editing of the manuscript, and its final contents. The authors declare that they have otherwise no known competing financial interests or personal relationships that could have appeared to influence the work reported in this paper.
